# Finerenone Improves Outcomes in Patients With Heart Failure With Mildly Reduced or Preserved Ejection Fraction Irrespective of Age: A Prespecified Analysis of FINEARTS-HF

**DOI:** 10.1161/CIRCHEARTFAILURE.124.012437

**Published:** 2024-09-29

**Authors:** Misato Chimura, Mark C. Petrie, Morten Schou, Felipe A. Martinez, Alasdair D. Henderson, Brian L. Claggett, Akshay S. Desai, Peter Kolkhof, Prabhakar Viswanathan, Andrea Lage, Carolyn S.P. Lam, Michele Senni, Sanjiv J. Shah, Katja Rohwedder, Katharina Mueller, Adriaan A. Voors, Faiez Zannad, Bertram Pitt, Muthiah Vaduganathan, Pardeep S. Jhund, Scott D. Solomon, John J.V. McMurray

**Affiliations:** 1 British Heart Foundation Cardiovascular Research Centre, University of Glasgow, United Kingdom (M.C., M.C.P., A.D.H., P.S.J., J.J.V.M.).; 2 Department of Cardiology, Herlev-Gentofte University Hospital, Hellerup, Denmark (M. Schou).; 3 Universidad Nacional de Córdoba, Argentina (F.A.M.).; 4 Cardiovascular Division, Brigham and Women’s Hospital, Harvard Medical School, Boston, MA (B.L.C., A.S.D., M.V., S.D.S.).; 5 Bayer AG, Berlin, Germany (P.K., P.V., A.L., K.R., K.M.).; 6 National Heart Centre Singapore and Duke-National University of Singapore (C.S.P.L.).; 7 University of Milano-Bicocca, Papa Giovanni XXIII Hospital, Bergamo, Italy (M. Senni).; 8 Northwestern University Feinberg School of Medicine, Chicago, IL (S.J.S.).; 9 University Medical Center Groningen, the Netherlands (A.A.V.).; 10 Université de Lorraine, Inserm Clinical Investigation Centre, University Hospital of Nancy, France (F.Z.).; 11 University of Michigan, School of Medicine, Ann Arbor (B.P.).

**Keywords:** age, finerenone, heart failure, hospitalization

## Abstract

**BACKGROUND:**

Finerenone improves outcomes in patients with heart failure and mildly reduced or preserved ejection fraction. It is important to understand the efficacy and safety of finerenone in these patients according to age.

**METHODS:**

The aim of this analysis was to evaluate the interaction between age and the efficacy and safety of finerenone in the FINEARTS-HF trial (Finerenone Trial to Investigate Efficacy and Safety Compared to Placebo in Patients With Heart Failure). A total of 6001 patients aged 40 to 97 years were stratified by quartile (Q1–Q4) of baseline age: Q1, 40 to 66 years (n=1581); Q2, 67 to 73 years (n=1587); Q3, 74 to 79 years (n=1421); and Q4, ≥80 years (n=1412). FINEARTS-HF evaluated the impact of age on the efficacy of finerenone with respect to the primary composite outcome of cardiovascular death and total (first and recurrent) heart failure events, including heart failure hospitalization or urgent heart failure event, along with secondary efficacy and safety outcomes.

**RESULTS:**

The incidence of primary outcomes increased with age. Finerenone reduced the risk of the primary outcome consistently across all age categories: rate ratio in Q1, 0.70 (95% CI, 0.53–0.92); Q2, 0.83 (95% CI, 0.64–1.07); Q3, 0.98 (95% CI, 0.76–1.26); and Q4, 0.85 (95% CI, 0.67–1.07); *P*_interaction_=0.27. Similarly, a consistent effect was observed for the components of the primary outcome. The mean increase in Kansas City Cardiomyopathy Questionnaire-total symptom score from baseline to 12 months was greater with finerenone than placebo, with a consistent effect across all age categories: mean placebo-corrected change in Q1, 2.87 (95% CI, 1.09–4.66); Q2, 1.24 (95% CI, −0.59 to 3.07); Q3, 0.94 (−0.98 to 2.86); and Q4, 1.24 (−0.90 to 3.38); *P*_interaction_=0.50. Adverse events were similar across all age categories. The odds of experiencing hypotension, elevated creatinine, or hyperkalemia (increased) or hypokalemia (decreased) related to finerenone did not differ by age.

**CONCLUSIONS:**

In the FINEARTS-HF trial, finerenone reduced the primary outcome and components of the primary outcome and improved symptoms across a wide age spectrum. In addition, finerenone was safe and well-tolerated, irrespective of age.

**REGISTRATION:**

URL: https://www.clinicaltrials.gov; Unique identifiers: NCT04435626 and EudraCT 2020-000306-29.

**WHAT IS NEW?**

- In the FINEARTS-HF trial (Finerenone Trial to Investigate Efficacy and Safety Superior to Placebo in Patients With Heart Failure), finerenone improved clinical outcomes and alleviated heart failure symptoms in 6001 patients with heart failure with mildly reduced and preserved ejection fraction.
- The efficacy and safety of finerenone were consistent across the age spectrum studied (40–97 years).

**WHAT ARE THE CLINICAL IMPLICATIONS?**

- The benefits of finerenone are consistent across all age groups, including patients aged ≥80 years.
- Hypotension, elevated creatinine, and hyperkalemia were more common with finerenone and hypokalemia less common with finerenone, but these differences between finerenone and placebo did not vary according to age.
- Advanced age, in itself, should not be a reason to withhold finerenone in patients with mildly reduced and preserved ejection fraction.

The prevalence of heart failure with mildly reduced and preserved ejection fraction (HFmrEF/HFpEF) is increasing, and it is projected that HFmrEF/HFpEF will soon surpass heart failure (HF) with reduced ejection fraction to become the predominant HF phenotype globally.^1,2^ This trend is primarily driven by aging populations worldwide, and in the United States alone, the population aged ≥80 years increased from 4.1 million in 1971 to 13.1 million in 2020, with this change expected to continue or accelerate.^3^ Consequently, identifying effective treatments for HFmrEF/HFpEF, particularly in the elderly, to reduce worsening HF events and improve health status has emerged as an important contemporary therapeutic challenge.^1,4–6^ This challenge is amplified by concerns that older patients with HFmrEF/HFpEF are often frail and have multiple comorbidities leading to polypharmacy. These considerations, coupled with differing pharmacodynamics and pharmacokinetics in older compared with younger people, raise further concerns that treatments may be less well-tolerated in older adults. Therefore, it is crucial to also evaluate the safety of incorporating new medications into the existing therapeutic regimens of these patients.^7^ Without an in-depth understanding of the efficacy and tolerability of novel therapies, there is a significant risk of their underuse, as has been repeatedly observed for many valuable treatments in older individuals.^8–10^

In the FINEARTS-HF trial (Finerenone Trial to Investigate Efficacy and Safety Superior to Placebo in Patients With Heart Failure), the effects of the selective nonsteroidal mineralocorticoid receptor antagonist finerenone were compared with those of placebo in patients with HFmrEF/HFpEF. Among the 6001 participants analyzed, finerenone added to background therapy reduced the risk of cardiovascular death and total (first and recurrent) HF events compared with placebo.^11^ FINEARTS-HF enrolled patients with a wide range of ages (40–97 years), and, notably, 64% of trial participants were aged ≥70 years and 24% were ≥80 years of age. In this prespecified analysis, we examined the efficacy and safety of finerenone compared with placebo, stratified by age.

## Methods

### FINEARTS-HF Trial Design and Objectives

FINEARTS-HF was a multicenter, prospective, randomized, double-blind, event-driven trial that examined the efficacy and safety of finerenone compared with placebo in patients with HFmrEF/HFpEF. The design, baseline characteristics, and primary results are published.^11–13^ Key inclusion criteria were age ≥40 years, symptomatic HF in New York Heart Association (NYHA) functional class II to IV, treatment with a diuretic for ≥30 days before randomization, and a left ventricular ejection fraction (LVEF) ≥40% with evidence of structural heart disease (either left atrial enlargement or left ventricular hypertrophy) measured within 12 months of screening. Patients were also required to have elevated levels of NT-proBNP (N-terminal pro-B-type natriuretic peptide) >300 pg/mL or BNP (B-type natriuretic peptide) >100 pg/mL for those in sinus rhythm, or NT-proBNP >900 pg/mL or BNP >300 pg/mL for those in atrial fibrillation. These measurements were to be taken within 90 days for patients with a recent worsening HF event within 90 days before randomization, or within 30 days before randomization for those without a recent worsening HF event. Both ambulatory and hospitalized patients were eligible for enrollment. Patients with prior LVEF <40% with subsequent improvement to ≥40% were also eligible for enrollment provided that ongoing HF symptoms were present and all other inclusion criteria were satisfied. Key exclusion criteria were estimated glomerular filtration rate (eGFR) <25 mL/(min·1.73 m^2^), serum potassium >5.0 mmol/L at screening or randomization, or symptomatic hypotension with mean systolic blood pressure <90 mm Hg at screening or randomization. A complete list of exclusion criteria is provided in the design article.^12^ Eligible participants were randomized in a 1:1 ratio to finerenone or a matching placebo. The starting dose was 10 mg once daily in participants with an eGFR ≤60 mL/(min·1.73 m^2^) with a maximum maintenance dose of 20 mg once daily, whereas the starting dose was 20 mg once daily if the eGFR was >60 mL/(min·1.73 m^2^) with a maximum maintenance dose of 40 mg once daily. Ethics committees for the 653 participating institutions in 37 countries approved the protocol, and all patients gave written consent. The corresponding author had full access to all the trial data and takes responsibility for its integrity and the data analysis. Trial data will be made available by the sponsor, Bayer, in accordance with their data sharing policy.

### Trial Outcomes

The primary outcome was the composite of total (first and recurrent) HF events, including HF hospitalization or an urgent HF event, and cardiovascular death. The secondary outcomes included total HF events (we also examined cardiovascular death and cardiovascular death or a first HF event); improvement in NYHA functional class from baseline to 12 months; change from baseline to 6, 9, and 12 months in the Kansas City Cardiomyopathy Questionnaire-total symptom score (KCCQ-TSS); time to first occurrence of the composite renal end point (defined as a sustained decrease in eGFR ≥50% relative to baseline over at least 4 weeks, or a sustained eGFR decline <15 mL/[min·1.73 m²], or the initiation of dialysis or renal transplantation); and time to all-cause death. Due to the small number of events for the composite renal end point, this end point was not examined in this subgroup analysis. The prespecified safety outcomes included incidents of hyperkalemia (defined as serum potassium >5.5 or >6.0 mmol/L), hypokalemia (defined as serum potassium <3.5 mmol/L), elevation of serum creatine (defined as serum creatinine ≥2.5 or ≥3.0 mg/dL), and hypotension (defined as systolic blood pressure <100 mm Hg).

### Statistical Analysis

Between 2020 and 2023, a total of 7463 patients from 37 countries were screened, with 6001 patients ultimately analyzed. Participants were stratified by quartile of baseline age: 40 to 66, 67 to 73, 74 to 79, and ≥80 years. Baseline characteristics were summarized as frequencies with percentages for categorical variables, means with SDs for normally distributed continuous variables, and medians with interquartile ranges for non-normally distributed continuous variables. Differences in baseline characteristics were assessed using the Cochran-Armitage trend test for binary variables, the Cochran-Mantel-Haenszel test for categorical variables, and the Jonckheere-Terpstra test for continuous variables. The Poisson regression model with robust SEs was used to calculate the incidence rate of events by age quartile and the rate of specific causes of death across the age spectrum. To compare the effects of finerenone versus placebo according to age, time-to-event data were evaluated using Kaplan-Meier curves and Cox proportional-hazards models, with treatment assignment as a fixed effect and region and baseline LVEF (<60% or ≥ 60%) as stratification factors, and hazard ratios with 95% CIs were reported. Total (first and recurrent) events were evaluated using Nelson-Aalen cumulative hazard curves and rate ratios with 95% CIs semiparametric proportional rates models,^14^ with adjustments and stratification for the same covariates mentioned above. Additionally, hazard ratios and rate ratios were adjusted for baseline variables (sex, heart rate, systolic blood pressure, body mass index, NT-proBNP [log], eGFR, NYHA functional class III/IV, LVEF, myocardial infarction, diabetes, history of atrial fibrillation, and history of HF hospitalization). The effect of finerenone versus placebo across the range of age as a continuous variable (2.5th–97.5th percentiles) was modeled using restricted cubic splines with 3 knots. The difference in the incidence rate of the primary composite outcome and total HF events across the range of age was estimated using predictions from a Poisson model (that included an offset variable to account for the differential follow-up) with robust SEs adjusted for treatment effect and an restricted cubic splines of age with 3 knots.

The change in KCCQ-TSS from baseline to 12 months was analyzed using a linear regression of change in month 12 KCCQ-TSS adjusted for baseline KCCQ-TSS, geographic region, and baseline LVEF strata. We computed least squares estimates of the mean change by treatment group at 12 months and the difference between treatment groups within each age quartile. In addition, the proportion of patients with improvement in NYHA functional class from baseline to 12 months was evaluated using logistic regression models, adjusted for treatment assignment and stratification levels, and odds ratios with 95% CIs were reported. In addition, odds ratios adjusted for the variables mentioned above were also reported. The incidence of safety end points was estimated using similar logistic regression models, and an interaction with age quartile was tested using a likelihood ratio test. Additional, post hoc, exploratory analyses were conducted on elderly patients, specifically those aged 75 to 79, 80 to 84, and >85 years. All statistical analyses were conducted using STATA, version 18 (College Station, TX), and a *P* value of <0.05 was considered nominally statistically significant.

## Results

Overall, 6001 patients aged between 40 and 97 years were randomized, with a mean age of 72 years. The age distribution (by quartile) was as follows: 1581 patients were aged 40 to 66 years, 1587 patients were 67 to 73 years, 1421 patients were 74 to 79 years, and 1412 patients were ≥80 years (Figure S1).

### Patient Characteristics According to Age Category

Older patients were predominantly female (Table 1). Most comorbidities, including atrial fibrillation, stroke, and anemia, were more prevalent among older patients compared with younger patients (although coronary heart disease and diabetes were not, showing an opposite age-related gradient). Older patients also had higher NT-proBNP levels but lower body mass index, eGFR, and hemoglobin levels. Patients aged ≥80 years had the highest mean LVEF, with a greater proportion of LVEF ≥50% compared with the 40 to 66 years category (73% versus 51%). Median baseline KCCQ-TSS, KCCQ-overall summary score, and KCCQ-clinical summary score decreased with age, indicating that older patients had worse health status (Table 1). Consistent with this, NYHA functional class distribution was also worse in older compared with younger patients. Regarding background HF medications, older patients were less frequently treated with renin-angiotensin system blockers and beta-blockers compared with younger patients. The use of sodium-glucose cotransporter 2 inhibitors was low overall but similar across all age categories (Table 1).

**Table 1.**
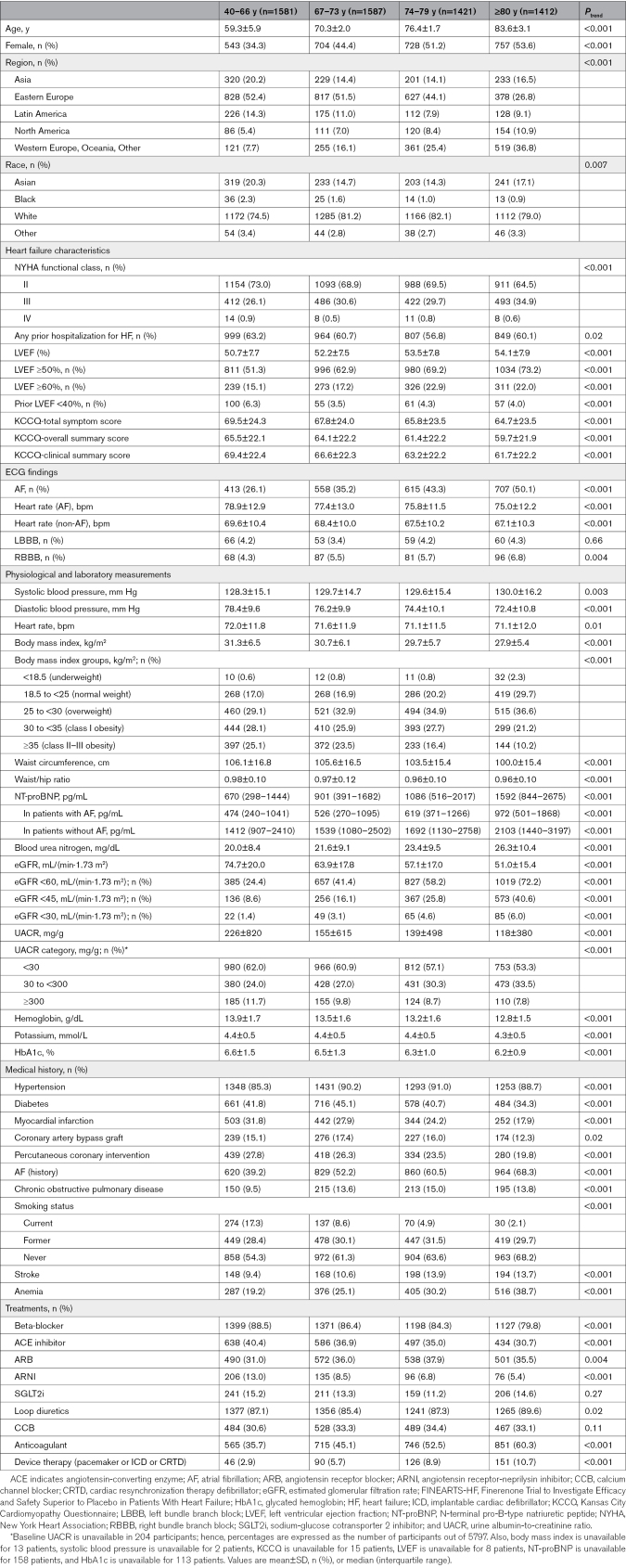
Baseline Characteristics According to Age Category (Quartile) in FINEARTS-HF

### Clinical Outcomes According to Age Category

**Figure 1.**
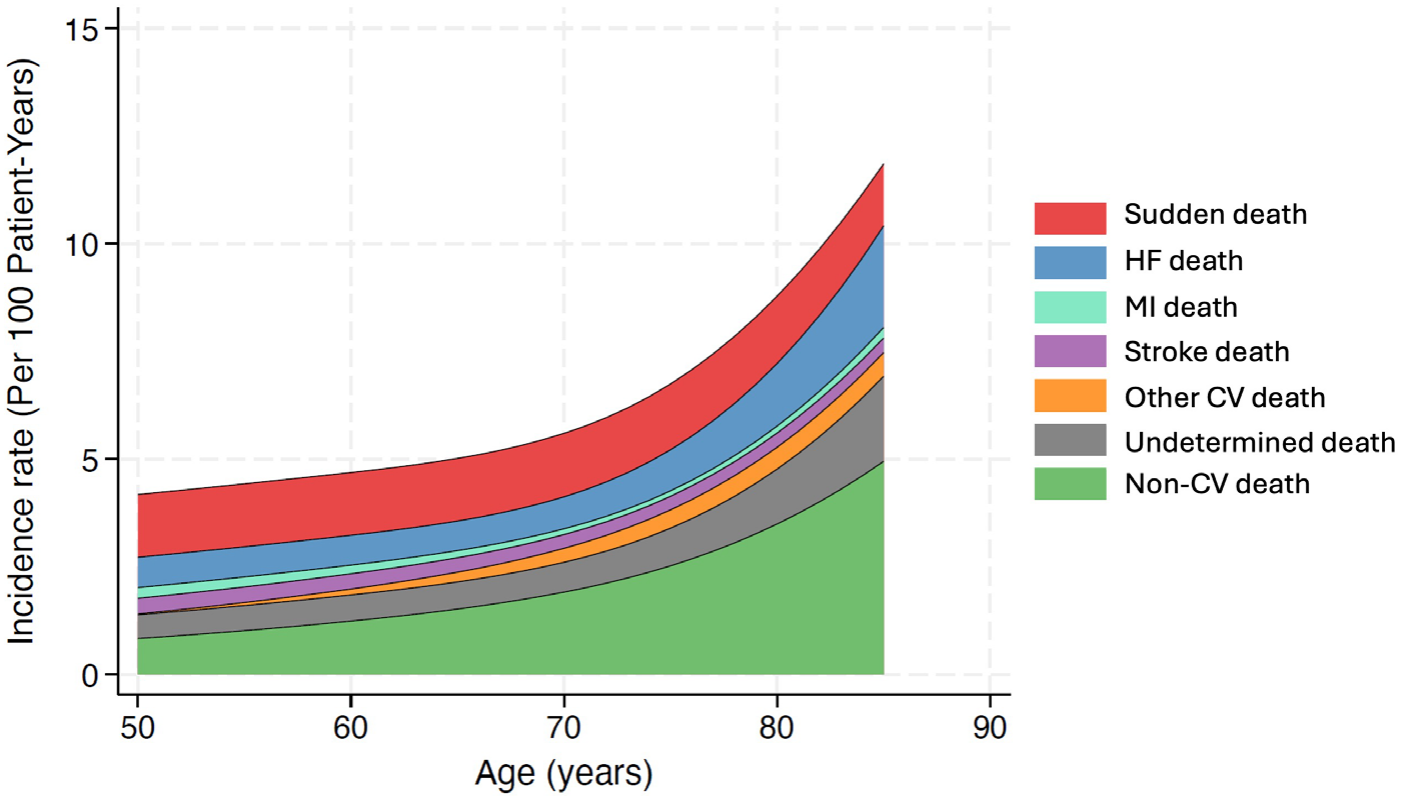
**Causes of death according to age in FINEARTS-HF (Finerenone Trial to Investigate Efficacy and Safety Superior to Placebo in Patients With Heart Failure).** The figure shows the variation in the incidence of different causes of death according to age. The incidence rate of each cause of death was assessed across the age range using a Poisson regression model where age was analyzed with restricted cubic splines incorporating 3 knots. CV indicates cardiovascular; HF, heart failure; and MI, myocardial infarction.

The incidence rate (per 100 patient-years) of the primary composite outcome increased with age: 40 to 66 years, 12.8 (95% CI, 11.1–14.7); 67 to 73 years, 14.9 (95% CI, 13.1–17.1); 74 to 79 years, 15.7 (95% CI, 13.9–17.8); and ≥80 years, 22.9 (95% CI, 20.3–25.8). Similar trends were observed for the components of the primary composite outcome, cardiovascular death or worsening HF analyzed as the time-to-first event, a first worsening HF event, and all-cause death (Table 2). The higher risk of worsening HF events associated with older age was largely eliminated when adjusted for other recognized prognostic variables. Further analysis of all-cause death showed that the causes of death varied across the spectrum of age, with a greatly increasing proportion of noncardiovascular deaths (and deaths from uncertain/unknown causes) with advancing age (Figure 1).

**Table 2.**
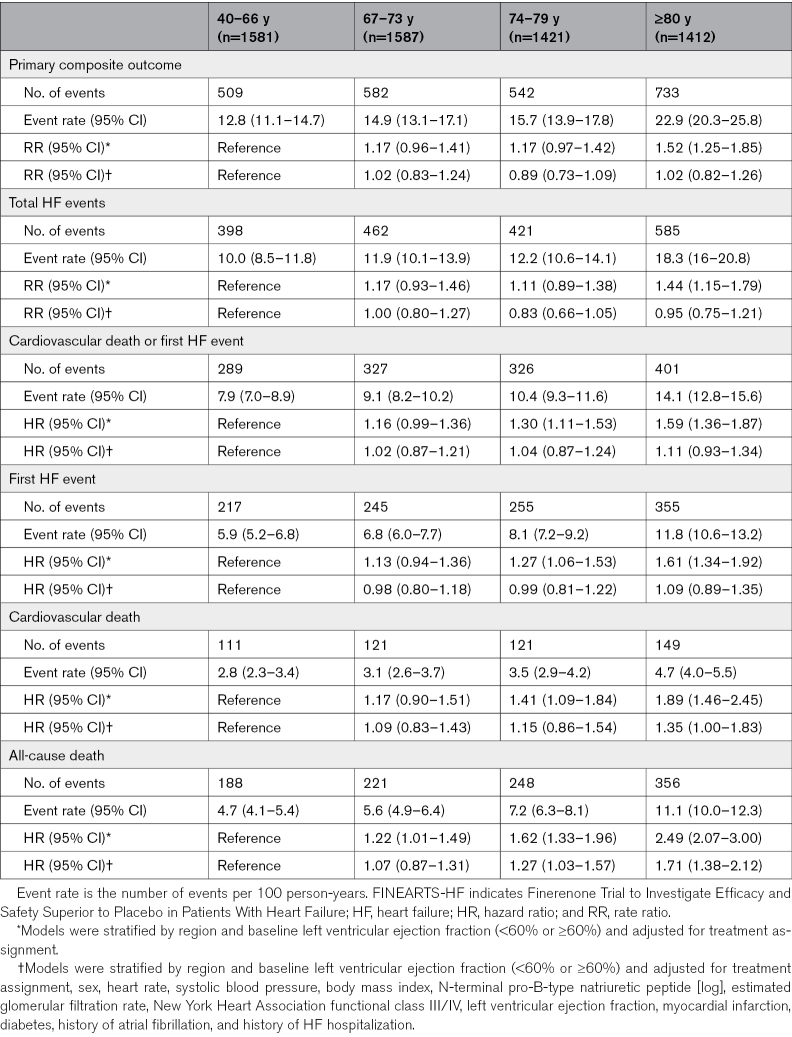
Outcomes According to Age Category (Quartiles) in FINEARTS-HF

### Effects of Finerenone According to Age Category

**Figure 2.**
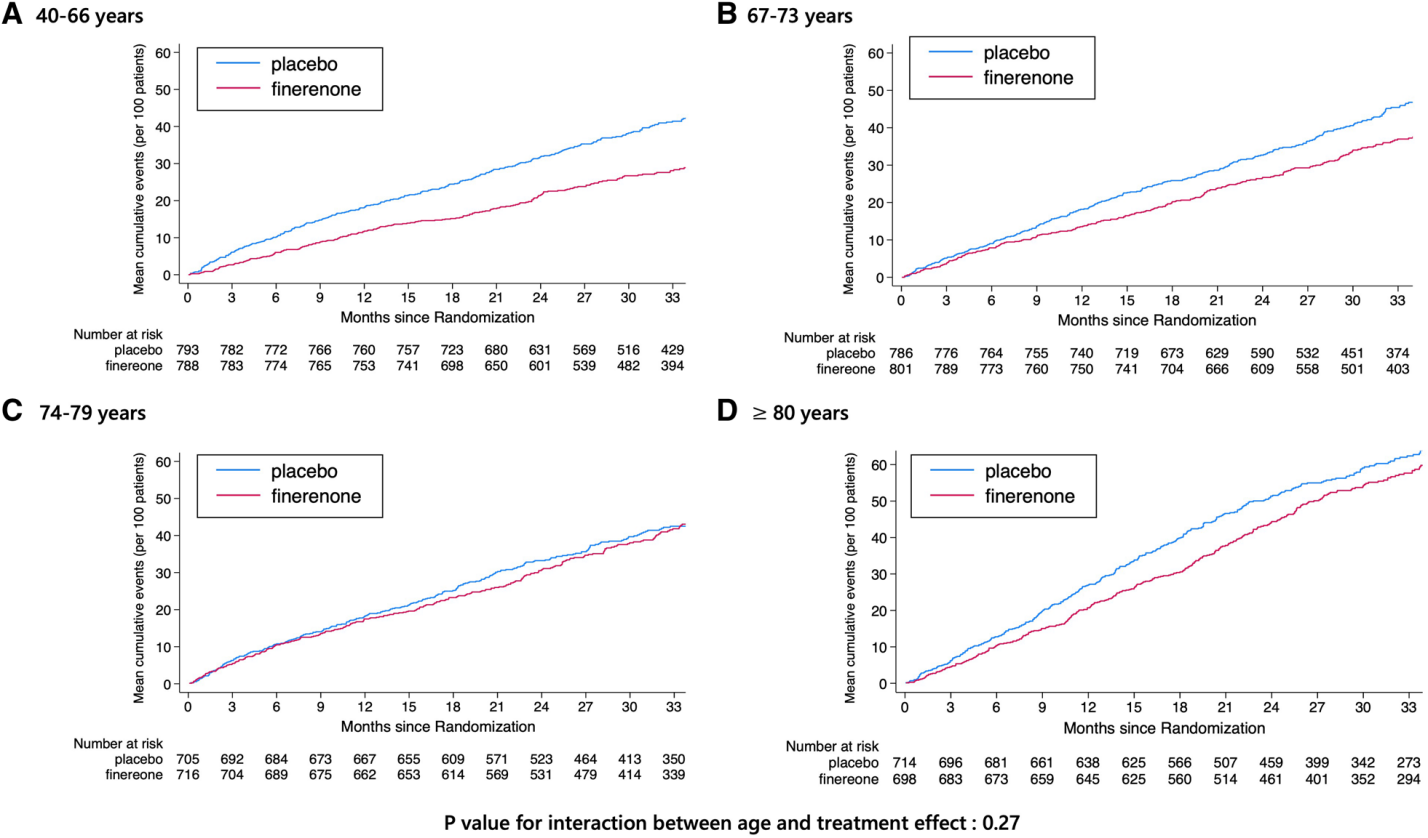
**Effect of finerenone on the primary composite outcome according to age category (quartiles) in FINEARTS-HF (Finerenone Trial to Investigate Efficacy and Safety Superior to Placebo in Patients With Heart Failure).** The figures show the Nelson-Aalen estimate of the cumulative hazard for the primary composite end point according to age categorized by quartile: 40 to 66 years (**A**), 67 to 73 years (**B**), 74 to 79 years (**C**), and ≥80 years (**D**). The blue solid lines indicate the placebo group, and the red solid lines indicate the finerenone group.

**Figure 3.**
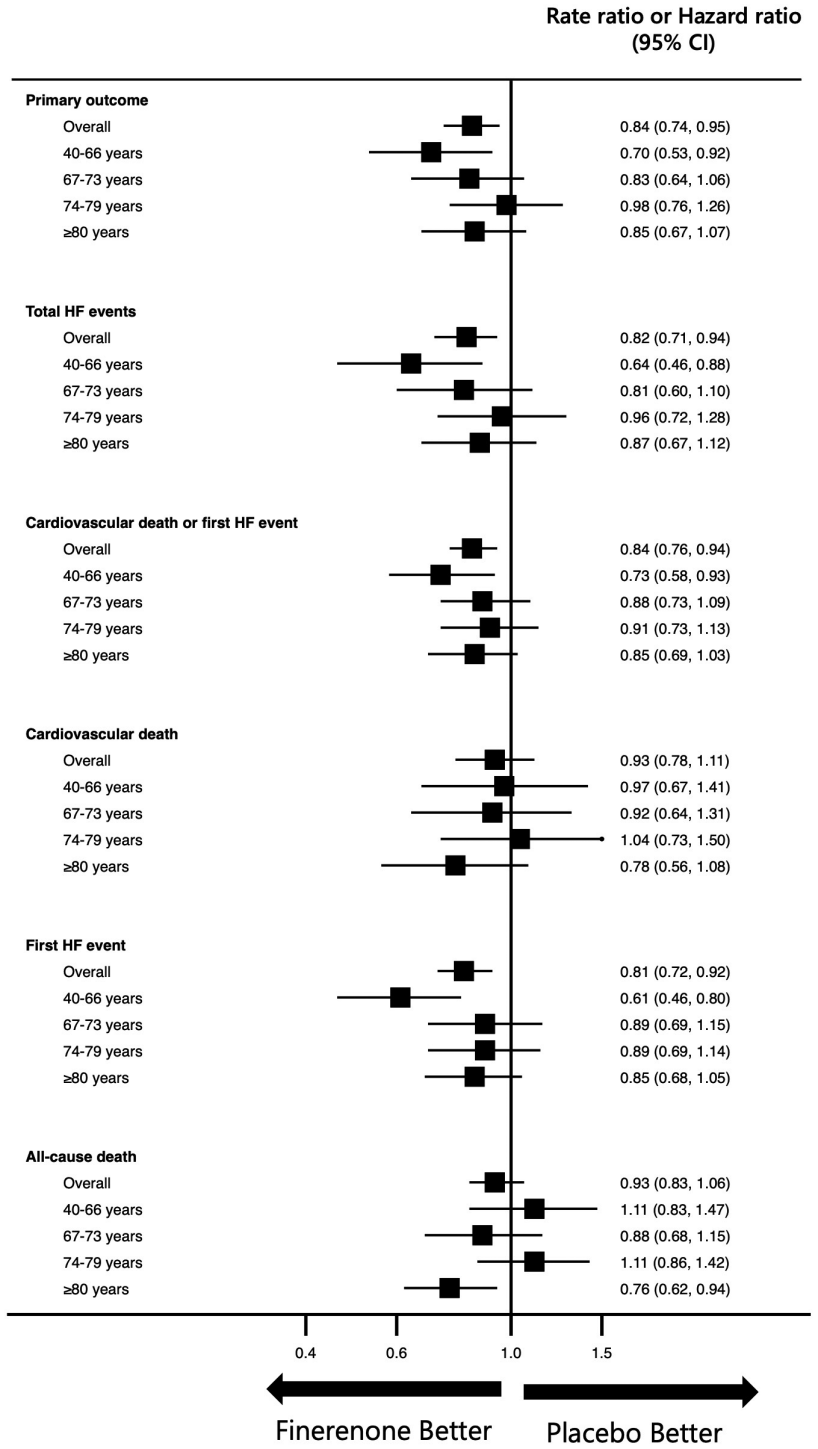
**Effects of finerenone on key outcomes according to age categories (quartiles) in FINEARTS-HF (Finerenone Trial to Investigate Efficacy and Safety Superior to Placebo in Patients With Heart Failure).** This figure shows the effect of finerenone, compared with placebo, on the primary composite point, total heart failure (HF) events, cardiovascular death or first HF event, cardiovascular death, first HF event, and all-cause death according to age category (defined by quartile of baseline age). The Lei-Wei-Yang-Ying (recurrent events) and Cox (time to first event) models are stratified by region and baseline left ventricular ejection fraction (<60% or ≥60%) and adjusted for treatment assignment. *P* values are for interaction between age groups and treatment effect.

Finerenone consistently reduced the risk of the primary outcome across all age categories, *P*_interaction_=0.27 (Table 3; Figures 2 and 3). Adjustments for key baseline differences across age groups and important prognostic variables did not change this finding (Table 3). The effect of finerenone was similarly consistent across the age spectrum for the components of the primary outcome, that is, total HF events (*P*_interaction_=0.22; Figure S2) and for cardiovascular death (*P*_interaction_=0.75; Figure S3). A similar pattern was seen for the composite of cardiovascular death or first HF event (*P*_interaction_=0.49), first HF event (*P*_interaction_=0.10), and all-cause death (*P*_interaction_=0.11; Table 3; Figure 3; Figures S4 through S6). An analysis conducted exclusively on patients aged ≥75 years demonstrated similar trends (Table S1).

**Table 3.**
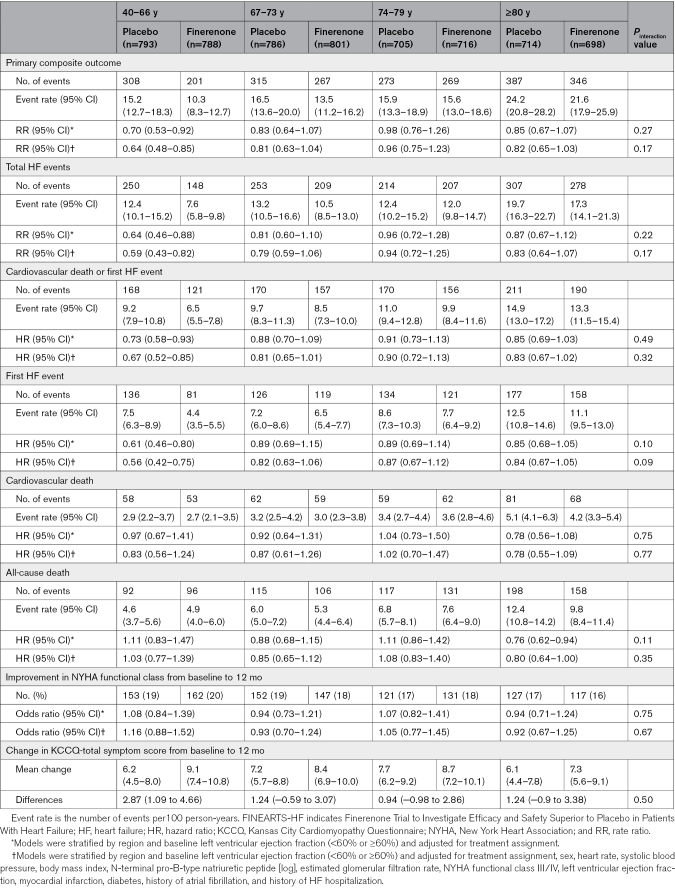
Effect of Randomized Treatment on Outcomes According to Age Category (Quartile) in FINEARTS-HF

**Figure 4.**
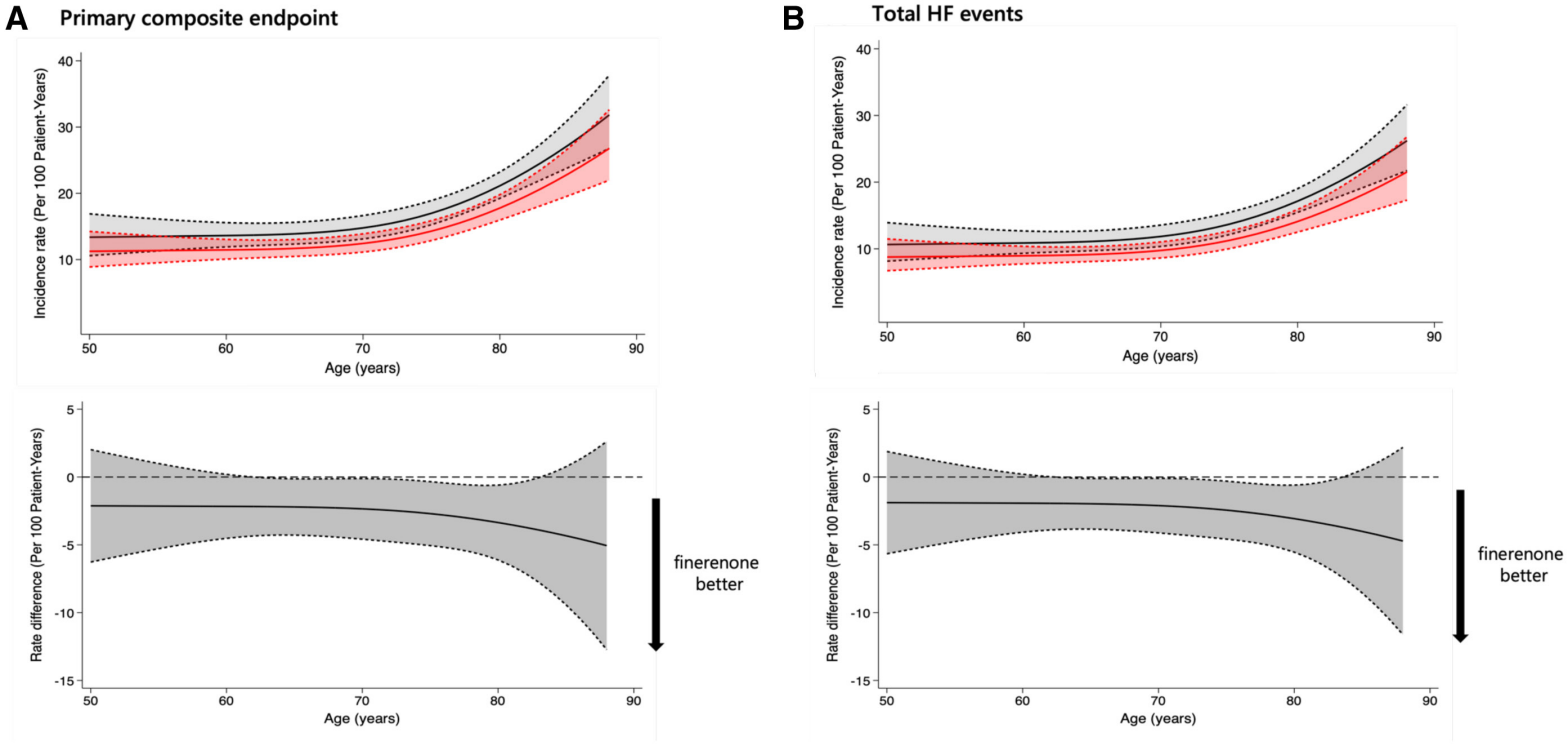
**Incidence of the primary and key secondary outcome across the spectrum of age (analyzed as a continuous variable) in FINEARTS-HF (Finerenone Trial to Investigate Efficacy and Safety Superior to Placebo in Patients With Heart Failure) and effect of finerenone compared with placebo.** The **top** panels show the associations between age and the incidence rate for the primary composite outcome (**A**) and total heart failure (HF) events (**B**). The gray lines represent the placebo group, and the red lines represent the finerenone group. The shaded areas represent the 95% CI. The **bottom** panels show the absolute benefits of finerenone (as a difference in event rates per 100 patient-years) across the range of ages for the same outcomes. The absolute difference in event rate is illustrated by the black line, and the shaded area represents the 95% CI. A rate difference <0 indicates a benefit of finerenone over placebo.

Analysis using age as a continuous rather than categorical variable gave similar findings for total HF events and cardiovascular death and total HF events (Figure 4) as well as for the other outcomes of cardiovascular death or first HF event, cardiovascular death, first HF event, and all-cause death (Figure S7). The absolute rate reduction with finerenone tended to be greater in older patients because of their higher event rates (Figure 4; Figure S7).

Mean KCCQ-TSS increased (improved) more between baseline and 12 months with finerenone than placebo, with a consistent benefit across age groups (*P*_interaction_=0.50; Table 3). We observed similar improvements, with no difference by age, in the other summary scores of KCCQ, the overall summary score, and the clinical summary score (Table S2). The odds of having a 5 point or greater improvement in the KCCQ-TSS, clinical summary score, and overall summary score were greater in the patients randomized to finerenone. Although the odds of improving were generally higher in the younger age groups, there was no evidence of interaction in the effect of finerenone, that is, we could find no difference in the effect on KCCQ scores by age.

NYHA functional class did not improve significantly with finerenone, compared with placebo, between baseline and 12 months, and this did not differ across the age categories (*P*_interaction_=0.75).

### Tolerability and Safety According to Age Category

The occurrence of prespecified laboratory safety measures and hypotension (defined as systolic blood pressure <100 mm Hg) according to age group are shown in Table 4. Older patients were more likely to have hypotension than younger patients, but there was no notable age gradient in the predefined laboratory safety measures. Patients randomized to finerenone were more likely to experience hypotension and hyperkalemia than those assigned to placebo, but less likely to experience hypokalemia. These between-treatment differences did not vary across age categories.

**Table 4.**
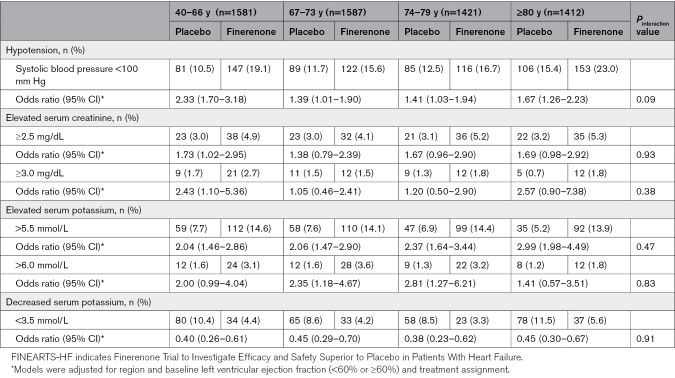
Laboratory Safety Assessments and Hypotension According to Age Category in FINEARTS-HF

Further exploratory analyses of these safety outcomes in the oldest patients showed a similar pattern (Table S3).

## Discussion

In the FINEARTS-HF trial, the nonsteroidal mineralocorticoid receptor antagonist finerenone was similarly effective in reducing the primary outcome of total worsening HF events, including HF hospitalizations or urgent HF visits, and cardiovascular death, across all age categories. Notably, 64% of trial participants were septuagenarians, and 24% were octogenarians and the efficacy of finerenone was consistent in these patients. Finerenone also improved health status compared with placebo, as evidenced by an increase in KCCQ-TSS, with no statistically significant interaction observed with age. Finally, the safety and tolerability profile of finerenone was favorable across all age categories.

The mean age in FINEARTS-HF (72 years) was similar to that reported in other recent HFmrEF/HFpEF trials, and the age-related trends in baseline characteristics observed were also consistent with those described in prior trials.^15–19^ Additionally, the majority of patients, regardless of age, were treated with renin-angiotensin-aldosterone system inhibitors, beta-blockers, and diuretics. Notably, older patients had higher baseline prevalence of atrial fibrillation, stroke, and anemia, as well as higher baseline NT-proBNP levels and lower eGFR and hemoglobin, variables known to be associated with worse outcomes.^18–21^ Consistent with this, the rates of all trial end points were highest in the oldest age groups, although, interestingly, the excess risk for worsening HF events in older age groups was substantially attenuated or even eliminated by adjustment for recognized prognostic variables, suggesting age alone makes only a small contribution to several of the worse outcomes in older patients with HFmrEF/HFpEF. This was not the case for mortality, especially all-cause mortality, possibly because of the larger contribution of noncardiovascular death to overall mortality in older patients.

The benefit of finerenone on the primary outcome was driven by a reduction in worsening HF events with no significant benefit on cardiovascular mortality. More importantly, we found that the benefit of finerenone, expressed as a relative risk reduction, was consistent across all age categories, with a greater absolute risk reduction in older patients. This highlights the potential risk-treatment paradox commonly identified in older patients who often have more to gain from therapies because of their higher baseline risk yet a lower probability of being prescribed such treatments.^22,23^

An additional therapeutic objective in HF is to alleviate symptom burden, enhance physical function, and improve health-related quality of life, thereby improving the patient’s overall health status. In the FINEARTS-HF trial, the increase in KCCQ-TSS was greater with finerenone compared with placebo, with a consistent increase observed across all age groups, including in the oldest patients. The mean overall increase in KCCQ-TSS was relatively small, but this may reflect generally mild symptoms of patients at baseline (69% NYHA functional class II), and the improvement in KCCQ-TSS was similar to that seen in other trials of pharmacotherapy for HFmrEF/HFpEF.^24–26^

The analysis of tolerability and safety was also favorable in the context of the aforementioned benefits of finerenone. For example, the proportion of patients ≥80 years exceeding a serum creatinine threshold of ≥3.0 mg/dL was 1.8% in the finerenone group versus 0.7% in the placebo group.

Additionally, while hypotension and hyperkalemia occurred more frequently with finerenone compared with placebo, across all age groups, there was no significant interaction between age and the effect of treatment on these measures. Kidney dysfunction, hyperkalemia, and hypotension are often particular concerns in elderly patients and lead to reluctance to initiate treatment (or discontinuation of treatment). Indeed, several recent trials have shown a markedly lower use of mineralocorticoid receptor antagonists in older compared with younger patients, for example, in the VICTORIA trial, mineralocorticoid receptor antagonists were used in 81% of patients <65 years versus 56% of patients ≥75 years.^27–29^

Many other studies have demonstrated lower usage of a range of guideline-recommended therapies in older patients with cardiovascular disease relative to younger patients, raising concerns about ageism in prescribing.^30–40^ Hopefully, the present findings are reassuring for the management of a growing and particularly high-risk population, which is often undertreated with effective therapies.

### Limitations

The interpretation of the findings from this trial must be considered in light of several limitations. Subdividing the patients by age resulted in smaller group sizes and fewer events, reducing the statistical power of these subgroup analyses. Despite these limitations, this trial represents one of the largest cohorts to date examining patients with HFmrEF or HFpEF across the age spectrum. There is always concern that patients in clinical trials are overly selected and that the efficacy and safety demonstrated in trials may not be representative of unselected real world populations. Interestingly, several recent studies have found that real world evidence has been largely consistent with trial findings.^41,42^

### Conclusions

Finerenone reduced the risk of HF events and cardiovascular death while also improving health-related quality of life and HF symptoms in patients with HFmrEF or HFpEF across the age spectrum. Additionally, finerenone was found to be safe and well-tolerated, irrespective of age.

## ARTICLE INFORMATION

### Sources of Funding

The FINEARTS-HF trial (Finerenone Trial to Investigate Efficacy and Safety Superior to Placebo in Patients With Heart Failure) was funded by Bayer.

### Disclosures

Dr Chimura received research grants and personal fees from Otsuka Pharma, Daiwa Foundation, and the Japan Research Foundation for Clinical Pharmacology. Dr Petrie reports, outside of the submitted work, grants or contracts from Boehringer Ingelheim, Roche, SQ Innovations, AstraZeneca, Novartis, Novo Nordisk, Medtronic, Boston Scientific, and Pharmacosmos; consulting fees from Akero, Applied Therapeutics, Amgen, AnaCardio, Biosensors, Boehringer Ingelheim, Novartis, AstraZeneca, Novo Nordisk, Abbvie, Bayer, Horizon Therapeutics, Foundry, Takeda, Cardiorentis, Pharmacosmos, Siemens, Eli Lilly, Vifor, New Amsterdam, Moderna, Teikoku, LIB Therapeutics, and 3R Lifesciences; and is Director of Global Clinical Trials Partners. Dr Schou reports other from Novo Nordisk, Novartis, AstraZeneca, and Boehringer outside the submitted work and reports personal fees from Bayer during the conduct of the study and personal fees from Alleviant, AstraZeneca, Boehringer Ingelheim, Edwards Lifesciences, Janssen, Novartis, Novo Nordisk, and Rivus outside the study. Dr Martinez reports personal fees from Bayer, during the conduct of the study. Dr Claggett has received personal consulting fees from Alnylam, Bristol Myers Squibb (BMS), Cardior, Cardurion, Corvia, CVRx, Eli Lilly, Intellia, and Rocket and has served on a data safety monitoring board for Novo Nordisk. Dr Desai has received institutional research grants (to Brigham and Women’s Hospital) from Abbott, Alnylam, AstraZeneca, Bayer, Novartis, and Pfizer, as well as personal consulting fees from Abbott, Alnylam, AstraZeneca, Bayer, Biofourmis, Boston Scientific, Medpace, Medtronic, Merck, Novartis, Parexel, Porter Health, Regeneron, River2Renal, Roche, Veristat, Verily, and Zydus. Drs Kolkhof, Lage, and Rohwedder and K. Mueller are employees of Bayer. Dr Viswanathan is a full-time employee of Bayer Pharmaceuticals. Dr Lam has received research support from NovoNordisk and Roche Diagnostics; has received consulting fees from Alleviant Medical, Allysta Pharma, AnaCardio AB, Applied Therapeutics, AstraZeneca, Bayer, Biopeutics, Boehringer Ingelheim, Boston Scientific, BMS, CardioRenal, CPC Clinical Research, Eli Lilly, Impulse Dynamics, Intellia Therapeutics, Ionis Pharmaceutical, Janssen Research and Development LLC, Medscape/WebMD Global LLC, Merck, Novartis, Novo Nordisk, Prosciento Inc, Quidel Corporation, Radcliffe Group Ltd, Recardio Inc, ReCor Medical, Roche Diagnostics, Sanofi, Siemens Healthcare Diagnostics, and Us2.ai; and is a cofounder and nonexecutive director of Us2.ai. Dr Senni has served on advisory boards, consultancy and honoraria for Novartis, Abbott, Merck, Vifor, AstraZeneca, Cardurion, Novo Nordisk, Bayer, and Boehringer Ingelheim. Dr Shah has received research grants from the National Institutes of Health (NIH; U54 HL160273, X01 HL169712, R01 HL140731, and R01 HL149423), the American Heart Association (24SFRNPCN1291224), AstraZeneca, Corvia, and Pfizer; and received consulting fees from Abbott, Alleviant, AstraZeneca, Amgen, Aria CV, Axon Therapies, Bayer, Boehringer Ingelheim, Boston Scientific, BMS, Cyclerion, Cytokinetics, Edwards Lifesciences, Eidos, Imara, Impulse Dynamics, Intellia, Ionis, Lilly, Merck, MyoKardia, Novartis, Novo Nordisk, Pfizer, Prothena, Regeneron, Rivus, Sardocor, Shifamed, Tenax, Tenaya, and Ultromics. Dr Voors’ employer received consultancy fees and/or research support from Adrenomed, Anacardio, AstraZeneca, Bayer AG, BMS, Boehringer Ingelheim, Corteria, EliLilly, Merck, Moderna, Novartis, Novo Nordisk, Roche Diagnostics, and SalubrisBio. Dr Zannad reports personal fees from 89Bio, Abbott, Acceleron, Applied Therapeutics, Bayer, Betagenon, Boehringer, BMS, CVRx, Cambrian, Cardior, Cereno Pharmaceutical, Cellprothera, CEVA, Inventiva, KBP, Merck, NovoNordisk, Owkin, Otsuka, Roche Diagnostics, Northsea, and USa2, having stock options at G3Pharmaceutical and equities at Cereno, Cardiorenal, and Eshmoun Clinical Research, and being the founder of Cardiovascular Clinical Trialists. Dr Pitt is a consultant for Bayer, Astra Zeneca, Boehringer Ingelheim, Lexicon, BMS, KBP Biosciences, Sarfez Pharmaceuticals, Pharmaceuticals, SQinnovations, G3 Pharmaceuticals, Sea Star Medical, Vifor, Prointel, and Brainstorm Medical; stock/stock options for KBP Biosciences, Sarfez Pharmaceuticals, Pharmaceuticals, SQinnovations, Sea Star Medical, Vifor, Prointel, and Brainstorm Medical; and US Patent 9931412-site specific delivery of eplerenone to the myocardium and US Patent pending 63/045,783 Histone modulating agents for the prevention and treatment of organ failure. Dr Vaduganathan has received research grant support, served on advisory boards, or had speaker engagements with American Regent, Amgen, AstraZeneca, Bayer AG, Baxter Healthcare, BMS, Boehringer Ingelheim, Chiesi, Cytokinetics, Fresenius Medical Care, Idorsia Pharmaceuticals, Lexicon Pharmaceuticals, Merck, Milestone Pharmaceuticals, Novartis, Novo Nordisk, Pharmacosmos, Relypsa, Roche Diagnostics, Sanofi, and Tricog Health; and participates on clinical trial committees for studies sponsored by AstraZeneca, Galmed, Novartis, Bayer AG, Occlutech, and Impulse Dynamics. Dr Jhund reports speakers’ fees from AstraZeneca, Novartis, Alkem Metabolics, ProAdWise Communications, and Sun Pharmaceuticals; advisory board fees from AstraZeneca, Boehringer Ingelheim, and Novartis; and research funding from AstraZeneca, Boehringer Ingelheim, Analog Devices Inc, and Roche Diagnostics. Dr Jhund’s employer, the University of Glasgow, has been remunerated for clinical trial work from AstraZeneca, Bayer AG, Novartis, and Novo Nordisk; and is the director of GCTP Ltd. Dr Solomon has received research grants from Alexion, Alnylam, AstraZeneca, Bellerophon, Bayer, BMS, Boston Scientific, Cytokinetics, Edgewise, Eidos, Gossamer, GSK, Ionis, Lilly, MyoKardia, the NIH/National Heart, Lung, and Blood Institute (NHLBI), Novartis, NovoNordisk, Respicardia, Sanofi Pasteur, Theracos, and Us2.ai; and has consulted for Abbott, Action, Akros, Alexion, Alnylam, Amgen, Arena, AstraZeneca, Bayer, Boeringer Ingelheim, BMS, Cardior, Cardurion, Corvia, Cytokinetics, Daiichi-Sankyo, GlaxoSmithKline (GSK), Lilly, Merck, Myokardia, Novartis, Roche, Theracos, Quantum Genomics, Janssen, Cardiac Dimensions, Tenaya, Sanofi Pasteur, Dinaqor, Tremeau, CellProThera, Moderna, American Regent, Sarepta, Lexicon, Anacardio, Akros, and Valo. Dr McMurray reports payments through Glasgow University from work on clinical trials, consulting, and grants from Amgen, AstraZeneca, Bayer, Cardurion, Cytokinetics, GSK, and Novartis, the British Heart Foundation, the NIH/NHLBI, Boehringer Ingelheim, SQ Innovations, and Catalyze Group; personal consultancy fees from Alynylam Pharmaceuticals, Amgen, AnaCardio, AstraZeneca, Bayer, Berlin Cures, BMS, Cardurion, Cytokinetics, Ionis Pharmaceuticals, Novartis, Regeneron Pharmaceuticals, and River 2 Renal Corp; personal lecture fees from Abbott, Alkem Metabolics, Astra Zeneca, Blue Ocean Scientific Solutions Ltd, Boehringer Ingelheim, Canadian Medical and Surgical Knowledge, Emcure Pharmaceuticals Ltd, Eris Lifesciences, European Academy of CME, Hikma Pharmaceuticals, Imagica Health, Intas Pharmaceuticals, JB Chemicals and Pharmaceuticals Ltd, Lupin Pharmaceuticals, Medscape/Heart.Org, ProAdWise Communications, Radcliffe Cardiology, Sun Pharmaceuticals, The Corpus, Translation Research Group, and Translational Medicine Academy; and Data Safety Monitoring Board for WIRB-Copernicus Group Clinical Inc. He is a director of Global Clinical Trial Partners Ltd. All other authors report no conflicts.

### Supplemental Material

Tables S1–S3

Figures S1–S7

## Supplementary Material
