# Supplementary material for: Finerenone Improves Outcomes in Patients With Heart Failure With Mildly Reduced or Preserved Ejection Fraction Irrespective of Age: A Prespecified Analysis of FINEARTS-HF
Source: Circ Heart Fail. 2024 Sep 29;17(11):e012437. doi: 10.1161/CIRCHEARTFAILURE.124.012437 (PMC11573060; doi:10.1161/CIRCHEARTFAILURE.124.012437)
Supplement: Supplementary file 1 [file hhf-17-e012437-s001.pdf]

SUPPLEMENTAL MATERIALS

Online Supplement for manuscript entitled: Finerenone improves outcomes in patients with heart failure with mildly reduced or preserved ejection fraction irrespective of age: A prespecified analysis of FINEARTS-HF

Tables: S1-S3

Figures: S1-S7

Table S1. Effect of randomized treatment on outcomes according to age group in FINEARTS-HF (including the very elderly)

	75 - 79 years (n = 1146)		80 - 84 years (n = 944)		≥ 85 years (n = 468)		Interaction P value
	Placebo (n = 558)	Finerenone (n = 588)	Placebo (n = 472)	Finerenone (n = 472)	Placebo (n = 242)	Finerenone (n = 226)	
**Primary composite outcome**							
Number of events	229	227	179	220	167	167	
Event rate (95% CI)	16.6 (13.6-20.2)	16.1 (13.2-19.7)	20.3 (16.6-24.8)	16.3 (13.3-19.9)	32.6 (25.9-41.0)	33.1 (24.2-45.3)	
RR (95% CI)*	0.97 (0.73-1.28)		0.76 (0.58-1.01)		0.99 (0.68-1.46)		0.49
RR (95% CI)**	0.94 (0.71-1.23)		0.76 (0.57-1.01)		0.89 (0.63-1.25)		0.61
**Total HF events**							
Number of events	180	183	173	138	134	140	
Event rate (95% CI)	13.2 (10.5-16.5)	12.9 (10.3-16.1)	15.9 (12.8-19.9)	12.5 (10.0-15.7)	26.2 (20.5-33.4)	27.8 (19.8-38.9)	
RR (95% CI)*	0.97 (0.71-1.33)		0.76 (0.56-1.03)		1.03 (0.69-1.55)		0.48
RR (95% CI)**	0.95 (0.70-1.29)		0.75 (0.55-1.03)		0.91 (0.63-1.30)		0.56
**Cardiovascular death or first HF event**							
Number of events	138	128	125	117	86	73	
Event rate (95% CI)	11.2 (9.5-13.3)	9.9 (8.3-11.8)	12.8 (10.7-15.3)	11.7 (9.8-14.0)	19.9 (16.0-24.8)	17.1 (13.4-21.7)	
HR (95% CI)*	0.89 (0.69-1.13)		0.88 (0.68-1.13)		0.81 (0.59-1.12)		0.86
HR (95% CI)**	0.87 (0.67-1.11)		0.88 (0.68-1.15)		0.77 (0.54-1.08)		0.73

**Cardiovascular death**							
Number of events	47	46	48	41	33	27	
Event rate (95% CI)	3.4 (2.6,-4.6)	3.2 (2.4-4.3)	4.4 (3.3-5.9)	3.7 (2.7-5.1)	6.4 (4.6-9.0)	5.4 (3.7-7.8)	
HR (95% CI)*	0.95 (0.63-1.44)		0.76 (0.50-1.16)		0.84 (0.50-1.41)		0.78
HR (95% CI)**	0.89 (0.58-1.36)		0.79 (0.51-1.23)		0.83 (0.47-1.47)		0.88
**First HF event**							
Number of events	108	103	100	93	77	65	
Event rate (95% CI)	8.8 (7.2-10.6)	7.9 (6.5-9.7)	10.2 (8.3-12.5)	9.3 (7.6-11.4)	17.8 (14.1-22.4)	15.2 (11.8-9.6)	
HR (95% CI)*	0.90 (0.68-1.18)		0.88 (0.67-1.17)		0.81 (0.58-1.14)		0.83
HR (95% CI)**	0.88 (0.66-1.17)		0.90 (0.67-1.21)		0.77 (0.54-1.10)		0.76
**All-cause death**							
Number of events	96	106	117	95	81	63	
Event rate (95% CI)	7.0 (5.7-8.5)	7.4 (6.1-9.0)	10.8 (9.0-12.9)	8.6 (7.0-10.5)	15.8 (12.8-19.5)	12.4 (9.8-15.8)	
HR (95% CI)*	1.06 (0.80-1.40)		0.76 (0.58-1.00)		0.77 (0.55-1.08)		0.17
HR (95% CI)**	1.00 (0.75-1.34)		0.79 (0.60-1.05)		0.82 (0.57-1.18)		0.38
**Improvement in NYHA functional class from baseline to 12 months**							
Number – no (%)	103 (18)	110 (18)	88 (18)	79 (16)	39 (16)	38 (16)	
Odds ratio (95% CI)*	1.02 (0.76-1.38)		0.90 (0.64-1.26)		1.06 (0.65-1.75)		0.77
Odds ratio (95% CI)**	0.94 (0.66-1.34)		0.92 (0.63-1.35)		0.84 (0.46-1.51)		0.98
**Change in KCCQ total symptom score from baseline to 12 months**							
Mean change	7.3 (5.6, 9.1)	8.4 (6.8, 10.1)	6.5 (4.4, 8.5)	7.6 (5.5, 9.6)	5.4 (2.1, 8.7)	6.7 (3.1, 10.3)	

Difference[#]	1.08 (-1.05, 3.22)	1.08 (-1.45, 3.62)	1.23 (-2.81, 5.28)	0.98

Abbreviations: CI, confidence interval, KCCQ, Kansas City Cardiomyopathy Questionnaire; HR, hazard ratio; HF, heart failure; NYHA, New York Heart Association; and RR, rate ratio

Event rate is the number of events per100 person-years.

* Models were stratified by region and baseline left ventricular ejection fraction (< 60% or ≥ 60%), and adjusted for treatment assignment.

**Models were stratified by region and baseline left ventricular ejection fraction (< 60% or ≥ 60%), and adjusted for treatment assignment, sex, heart rate, systolic blood pressure, body mass index, N-terminal pro-B-type natriuretic peptide [log], estimated glomerular filtration rate, NYHA functional class III/IV, left ventricular ejection fraction, myocardial infarction, diabetes mellitus, history of atrial fibrillation and history of HF hospitalization

[#] Linear regression model for change in KCCQ-total symptom score at month 12 adjusted for treatment, age quartile, baseline KCCQ total symptom score value, geographic region, and baseline left ventricular ejection fraction strata.

Table S2. Effect of randomized treatment on Kansas City Cardiomyopathy Questionnaire (KCCQ) scores according to age group in FINEARTS-HF

	40 - 66 years		67 - 73 years		74 - 79 years		≥ 80 years		Interaction P value
	Placebo (n = 793)	Finerenone (n = 788)	Placebo (n = 786)	Finerenone (n = 801)	Placebo (n = 705)	Finerenone (n = 716)	Placebo (n =714)	Finerenone (n =698)	
**Change in KCCQ total symptom score from baseline to 12 months**									
Mean change	6.2 (4.5, 8.0)	9.1 (7.4, 10.8)	7.2 (5.7, 8.8)	8.4 (6.9, 10.0)	7.7 (6.2, 9.2)	8.7 (7.2, 10.1)	6.1 (4.4, 7.8)	7.3 (5.6, 9.1)	
Difference	2.87 (1.09, 4.66)		1.24 (-0.59, 3.07)		0.94 (-0.98, 2.86)		1.24 (-0.9, 3.38)		0.50
Increase ≥ 5 points, n (%)	426 (57%)	454 (61%)	428 (58%)	425 (55%)	376 (56%)	384 (56%)	333 (50%)	319 (49%)	
Odds ratio (95% CI)*	1.21 (0.98, 1.49)		0.89 (0.72, 1.09)		1.01 (0.81, 1.25)		0.99 (0.80, 1.23)		0.24
Odds ratio (95% CI)**	1.25 (1.01, 1.55)		0.91 (0.74, 1.13)		1.02 (0.81, 1.27)		0.97 (0.78, 1.22)		0.23
**Change in KCCQ overall summary score from baseline to 12 months**									
Mean change	5.2 (3.6, 6.9)	7.7 (6.1, 9.4)	5.3 (3.9, 6.8)	6.3 (4.8, 7.7)	6.7 (5.2, 8.1)	6.9 (5.5, 8.3)	5.5 (3.9, 7.1)	5.5 (3.9, 7.1)	
Difference	2.50 (0.79, 4.21)		0.96 (-0.75, 2.67)		0.22 (-1.62, 2.06)		0.001 (-2.01, 2.02)		0.23
Increase ≥ 5 points, n (%)	364 (51%)	393 (56%)	351 (50%)	372 (51%)	312 (49%)	304 (48%)	284 (45%)	255 (42%)	
Odds ratio (95% CI)*	1.23 (1.00, 1.52)		1.03 (0.83, 1.26)		0.96 (0.77, 1.20)		0.90 (0.72, 1.13)		0.20
Odds ratio (95% CI)**	1.25 (1.01, 1.56)		1.06 (0.86, 1.32)		0.95 (0.75, 1.19)		0.90 (0.71, 1.13)		0.15
**Change in KCCQ clinical summary score from baseline to 12 months**									
Mean change	4.0 (2.4, 5.7)	6.4 (4.8, 8.0)	4.5 (3.1, 5.9)	5.5 (4.1, 6.9)	5.7 (4.3, 7.2)	6.6 (5.2, 8.1)	4.4 (2.8, 6.0)	4.4 (2.7, 6.0)	
Difference	2.34 (0.67, 4.02)		1.03 (-0.64, 2.71)		0.89 (-0.95, 2.73)		-0.05 (-2.06, 1.97)		0.34

Increase ≥ 5 points, n (%)	372 (52%)	402 (57%)	354 (51%)	364 (50%)	304 (48%)	312 (49%)	286 (45%)	259 (43%)	
Odds ratio (95% CI)*	1.24 (1.00, 1.53)		0.96 (0.78, 1.18)		1.06 (0.85, 1.32)		0.91 (0.73, 1.15)		0.23
Odds ratio (95% CI)**	1.27 (1.02, 1.57)		0.97 (0.78, 1.20)		1.03 (0.82, 1.30)		0.91 (0.72, 1.16)		0.27

* Models were stratified by region and baseline left ventricular ejection fraction (< 60% or ≥ 60%) and adjusted for treatment assignment. **Models were stratified by region and baseline left ventricular ejection fraction (< 60% or ≥ 60%), and adjusted for treatment assignment, sex, heart rate, systolic blood pressure, body mass index, N-terminal pro-B-type natriuretic peptide [log], estimated glomerular filtration rate, New York Heart Association functional class III/IV, left ventricular ejection fraction, myocardial infarction, diabetes mellitus, history of atrial fibrillation and history of heart failure hospitalization

Table S3. Tolerability of randomized treatment according to age group in FINEARTS-HF (including the very elderly)

	75 - 79 years (n = 1146)		80 - 84 years (n = 944)		≥ 85 years (n = 468)		Interaction P value
	Placebo	Finerenone	Placebo	Finerenone	Placebo	Finerenone	
**Hypotension – no (%)**							
Systolic blood pressure <100 mmHg	66 (12.3)	90 (15.9)	59 (12.8)	103 (23.1)	47 (20.5)	50 (22.8)	
Odds ratio (95% CI)*	1.33 (0.93-1.91)		2.28 (1.58-3.31)		1.01 (0.63-1.62)		0.02
**Elevated serum creatinine – no (%)**							
≥ 2.5 mg/dl	21 (3.9)	34 (6.1)	15 (3.3)	21 (4.8)	7 (3.1)	14 (6.4)	
Odds ratio (95% CI)*	1.54 (0.88-2.69)		1.58 (0.80-3.13)		1.95 (0.76-5.00)		0.86
≥ 3.0 mg/dl	9 (1.7)	11 (2.0)	4 (0.9)	7 (1.6)	1 (0.5)	5 (2.3)	
Odds ratio (95% CI)*	1.10 (0.45-2.71)		1.97 (0.57-6.83)		4.62 (0.52-40.76)		0.33
**Elevated serum potassium – no (%)**							
> 5.5 mmol/L	36 (6.7)	80 (14.2)	20 (4.4)	65 (14.7)	15 (6.7)	27 (12.3)	
Odds ratio (95% CI)*	2.47 (1.62-3.76)		3.77 (2.23-6.37)		2.02 (1.02-3.97)		0.31
> 6.0 mmol/L	6 (1.1)	18 (3.2)	6 (1.3)	9 (2.0)	2 (0.9)	3 (1.4)	
Odds ratio (95% CI)*	3.48 (1.35-8.99)		1.45 (0.51-4.15)		1.49 (0.22-10.07)		0.43
**Decreased serum potassium – no (%)**							
< 3.5 mmol/L	47 (8.8)	19 (3.4)	46 (10.1)	24 (5.4)	32 (14.4)	13 (5.9)	
Odds ratio (95% CI)*	0.36 (0.21-0.63)		0.51 (0.31-0.86)		0.37 (0.19-0.73)		0.64

Abbreviations: CI, confidence interval

*Models were adjusted for region and baseline left ventricular ejection fraction (< 60% or ≥ 60%), and treatment assignment.

Figure S1. Distribution of age in FINEARTS-HF

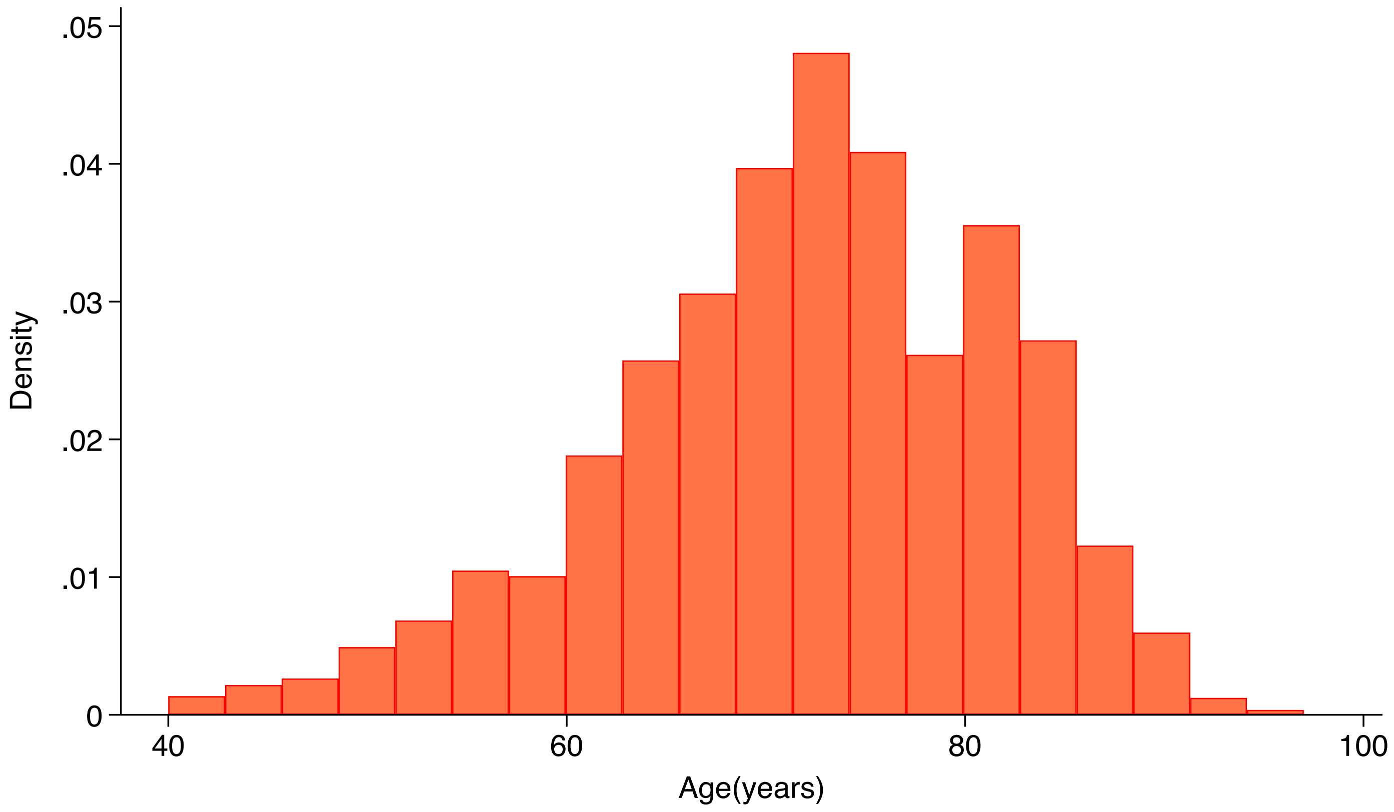

Figure S2. Cumulative incidence of total HF events according to age group

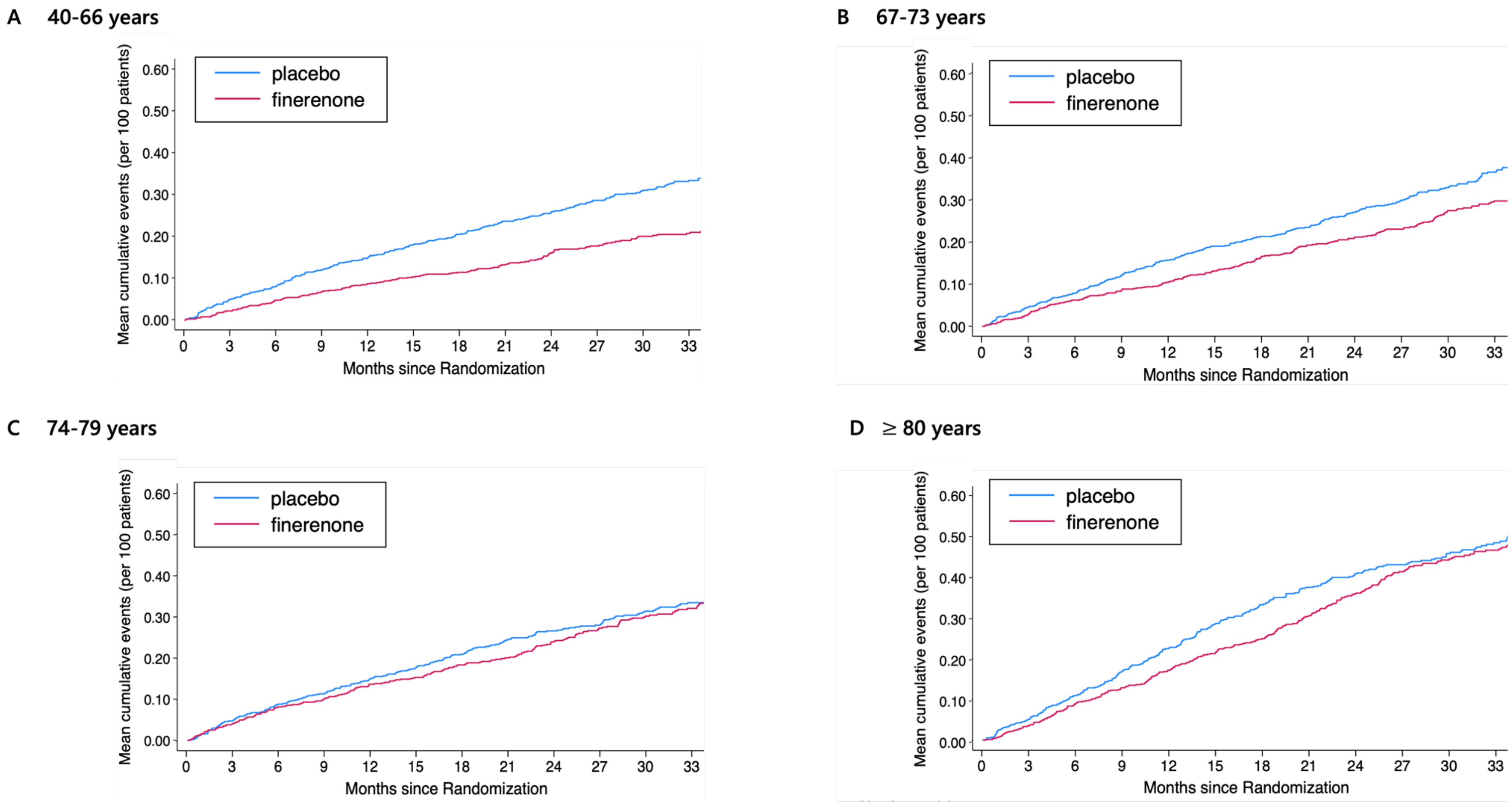

P value for interaction between age and treatment effect : 0.22

Figure S3. Cumulative incidence of cardiovascular death or first HF event according to age group

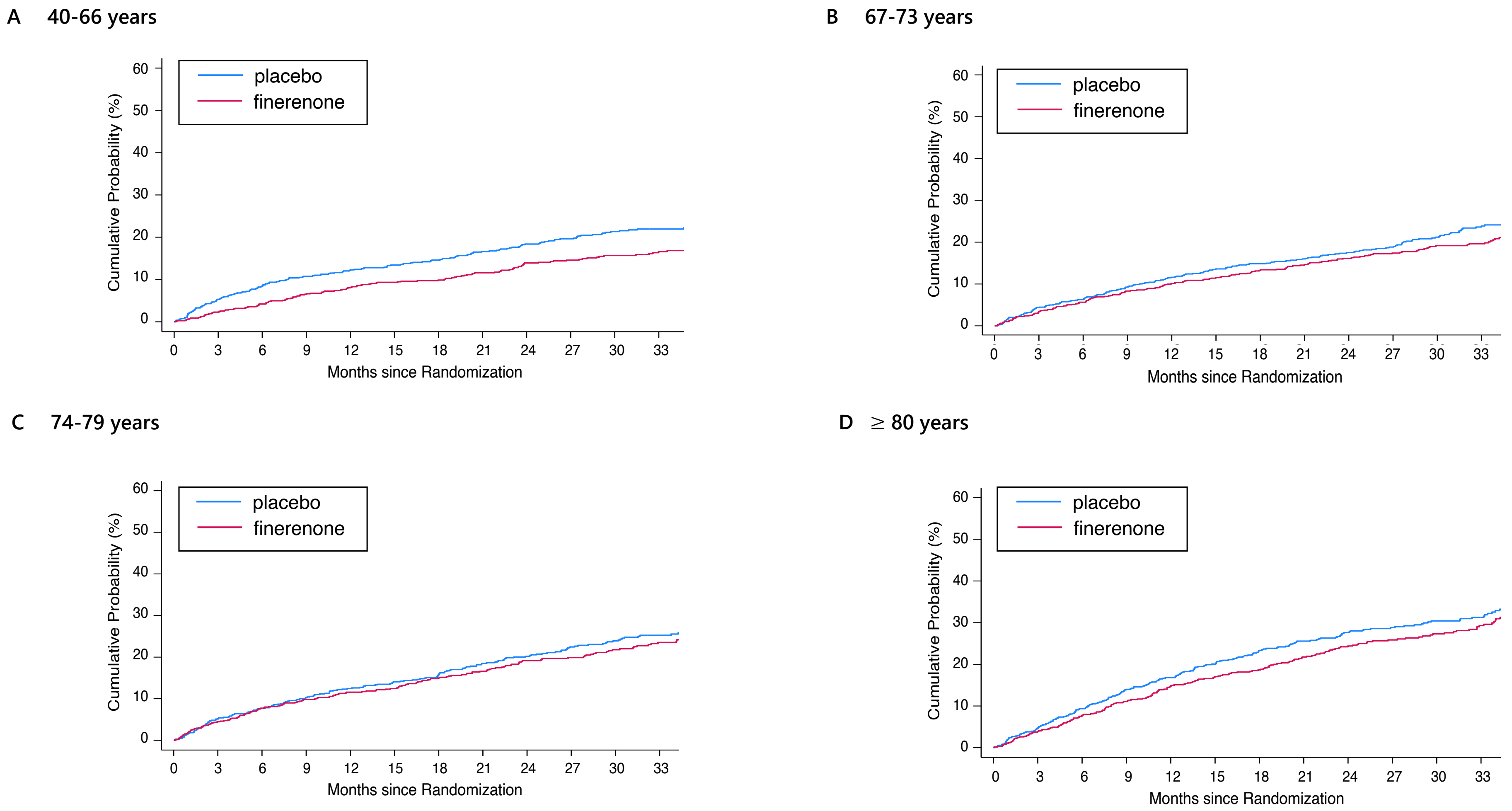

P value for interaction between age and treatment effect : 0.49

Figure S4. Cumulative incidence of cardiovascular death according to age group

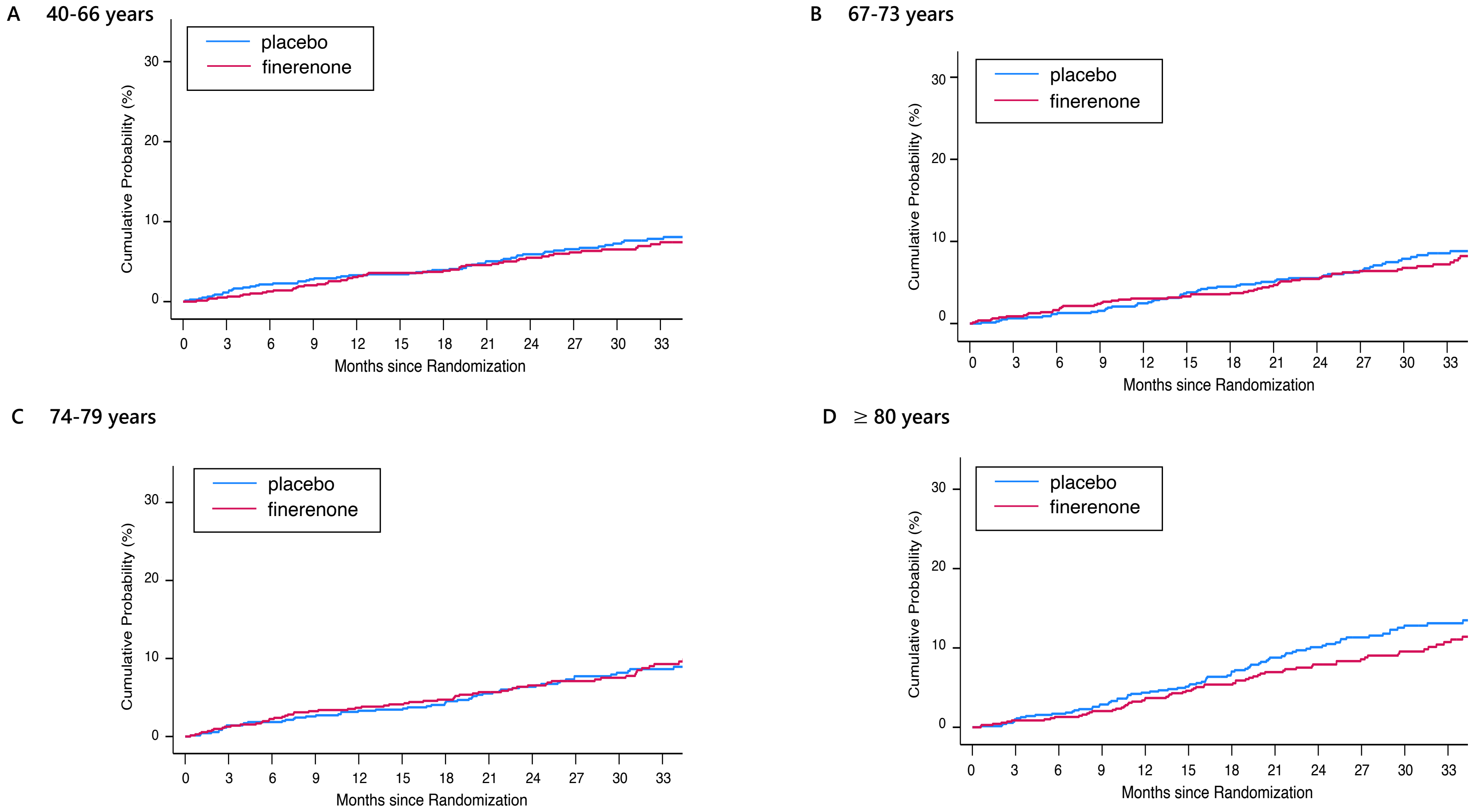

P value for interaction between age and treatment effect : 0.75

Figure S5. Cumulative incidence of first HF event (HF hospitalization or urgent HF event) according to age group

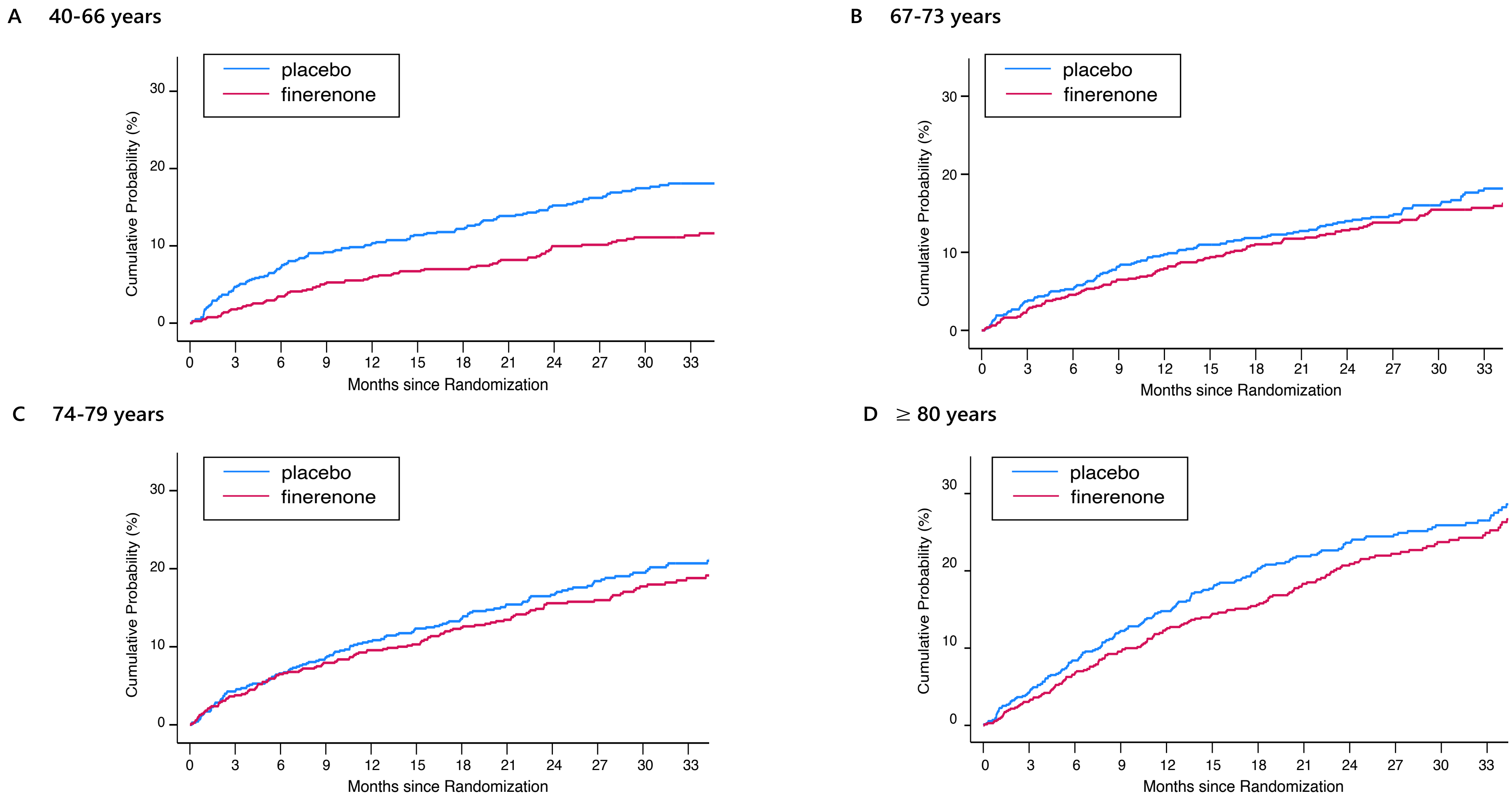

P value for interaction between age and treatment effect : 0.10

Figure S6. Cumulative incidence of all cause death according to age group

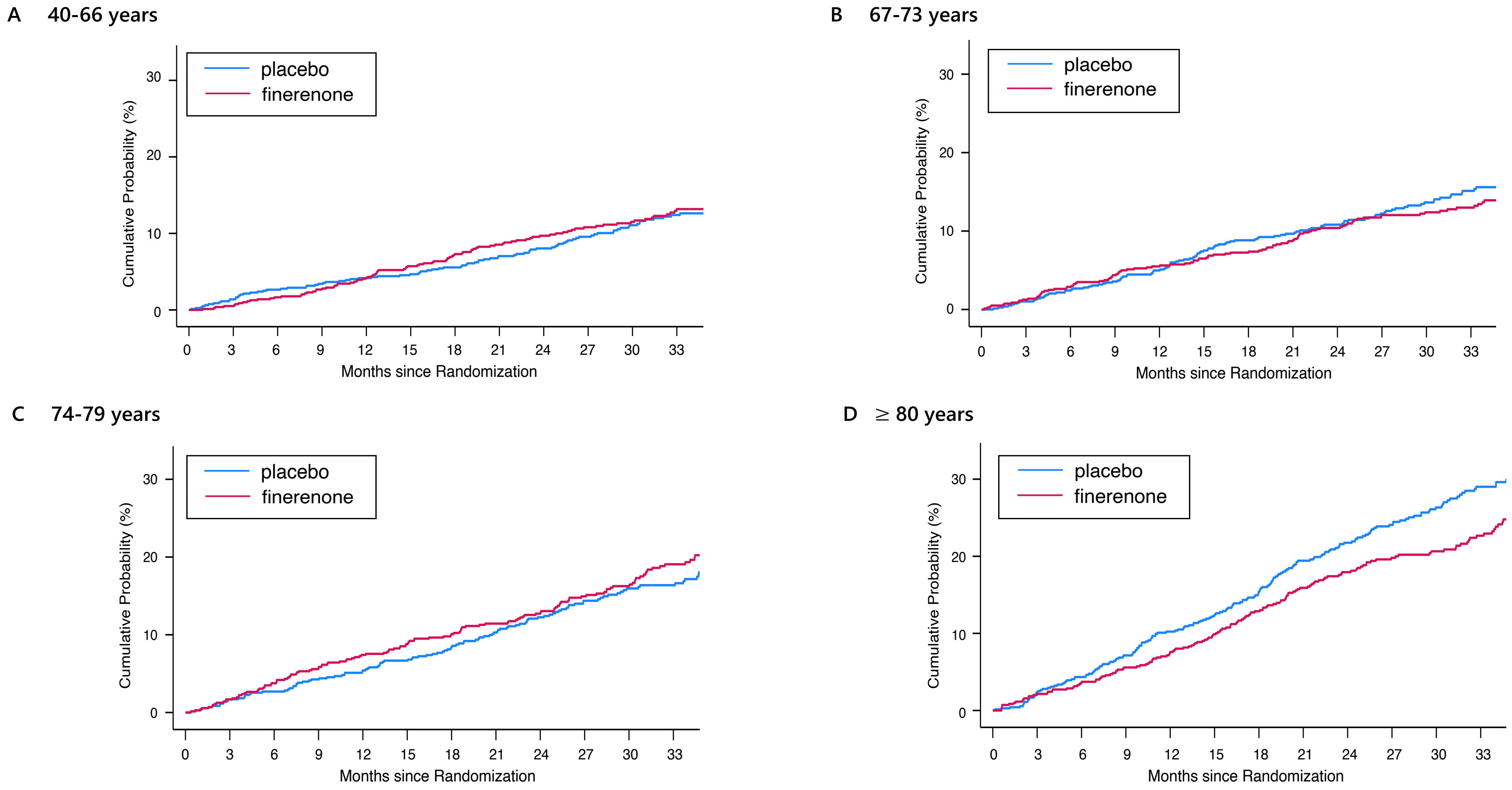

P value for interaction between age and treatment effect : 0.11

Figure S7. Incidence of cardiovascular death or first HF event, cardiovascular death, first HF event and all cause death across the spectrum of age (analyzed as a continuous variable) in FINEARTS-HF and effect of finerenone compared to placebo

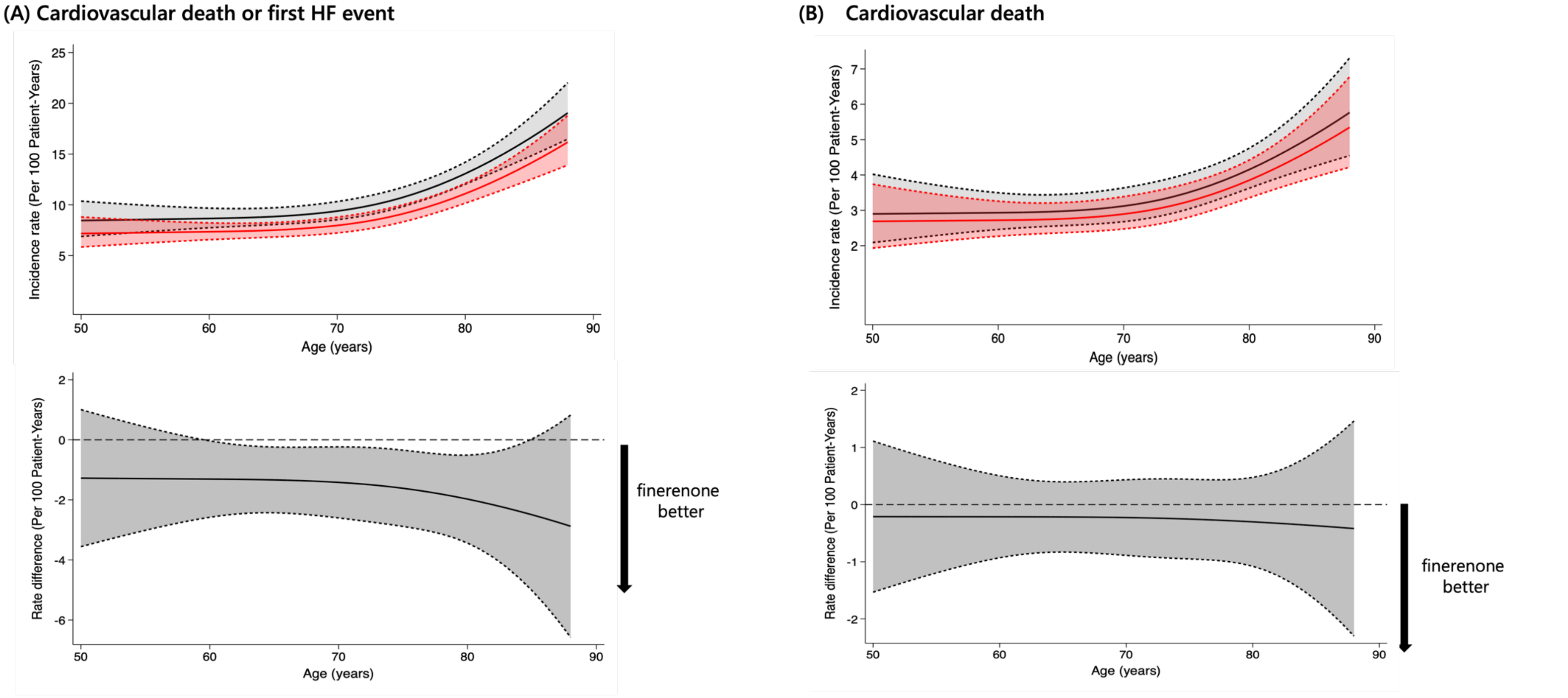

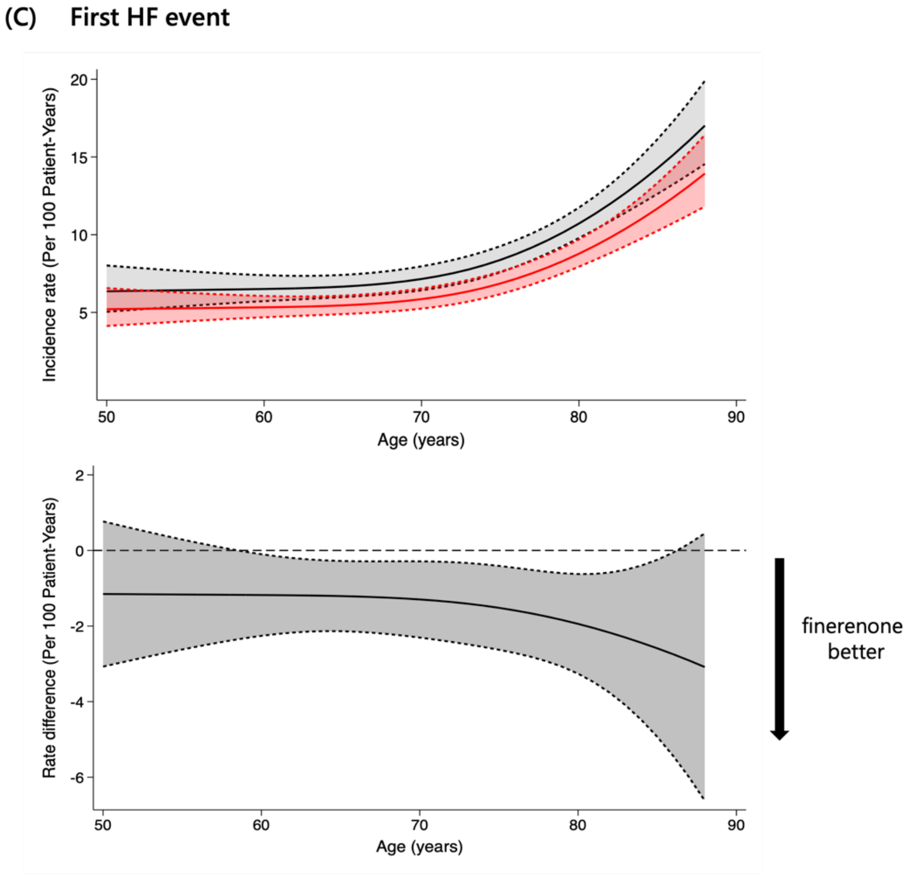

(C) First HF event
Incidence rate (Per 100 Patient-Years)
20
15
10
5
50
60
70
80
90
Age (years)
Rate difference (Per 100 Patient-Years)
2
0
-2
-4
-6
finerenone better

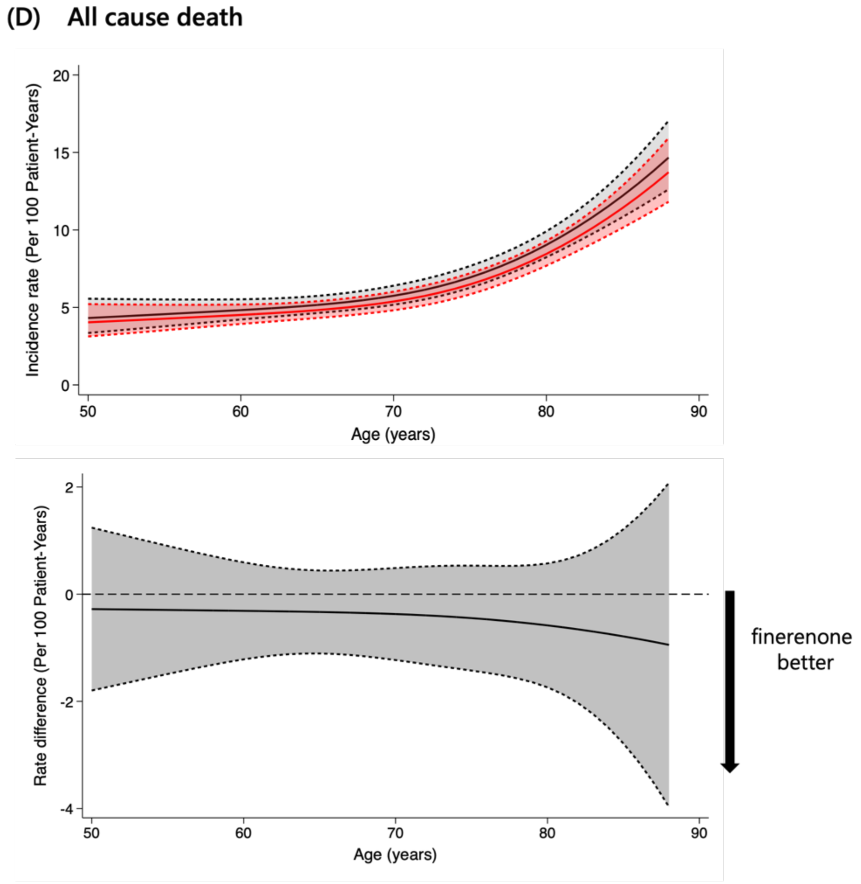

(D) All cause death
Incidence rate (Per 100 Patient-Years)
20
15
10
5
0
50
60
70
80
90
Age (years)
Rate difference (Per 100 Patient-Years)
2
0
-2
-4
finerenone better

Figure S1. Distribution of age in FINEARTS-HF

Figure S2. Cumulative incidence of total HF events according to age group

The figures show the cumulative incidence of total HF events according to age categorized by quartile: 40-66 years (Panel A), 67-73 years (Panel B), 74-79 years (Panel C), and ≥ 80 years (Panel D) (The blue solid line: placebo group, and the red solid line: finerenone group). Abbreviations: HF, heart failure

Figure S3. Cumulative incidence of cardiovascular death or first HF event according to age group

The figures show the cumulative incidence of cardiovascular death or first HF event according to age categorized by quartile: 40-66 years (Panel A), 67-73 years (Panel B), 74-79 years (Panel C), and ≥ 80 years (Panel D) (The blue solid line: placebo group, and the red solid line: finerenone group). Abbreviations: HF, heart failure

Figure S4. Cumulative incidence of cardiovascular death according to age group

The figures show the cumulative incidence of cardiovascular death according to age categorized by quartile: 40-66 years (Panel A), 67-73 years

(Panel B), 74-79 years (Panel C), and ≥ 80 years (Panel D) (The blue solid line: placebo group, and the red solid line: finerenone group).

Abbreviations: HF, heart failure

Figure S5. Cumulative incidence of first HF event (HF hospitalization or urgent HF event) according to age group

The figures show the cumulative incidence of first HF event (HF hospitalization or urgent HF event) according to age categorized by quartile: 40-66 years (Panel A), 67-73 years (Panel B), 74-79 years (Panel C), and ≥ 80 years (Panel D) (The blue solid line: placebo group, and the red solid line: finerenone group). Abbreviations: HF, heart failure

Figure S6. Cumulative incidence of all cause death according to age group

The figures show the cumulative incidence of all cause death according to age categorized by quartile: 40-66 years (Panel A), 67-73 years (Panel B), 74-79 years (Panel C), and ≥ 80 years (Panel D) (The blue solid line: placebo group, and the red solid line: finerenone group). Abbreviations: HF, heart failure

Figure S7. Incidence of cardiovascular death or first HF event, cardiovascular death, first HF event and all cause death across the spectrum of age (analyzed as a continuous variable) in FINEARTS-HF and effect of finerenone compared to placebo (A) Cardiovascular death or first HF event, (B) Cardiovascular death, (C) First HF event, and (D) All cause death Abbreviations: HF, heart failure
